# Influence factors for upper respiratory tract infection in Chinese rural children: A cross-sectional study

**DOI:** 10.3389/fped.2022.954363

**Published:** 2022-12-07

**Authors:** Bichen Wu, Shujuan Luo, Chang Xu, Ting Yang, Yanping Chen

**Affiliations:** Respiratory Department, Hunan Children's Hospital, Changsha, China

**Keywords:** influence factors, upper respiratory tract infection, China, rural children, CHNS

## Abstract

**Aim:**

The prevalence rate of upper respiratory tract infection (URTI) is high in children. Influencing factors for URTI have been reported in Chinese urban children, but those have not been explored in rural children. In China, children in the rural areas are a disadvantaged group. Therefore, this study aims to explore influencing factors for URTI in Chinese rural children.

**Methods:**

This is a cross-sectional study based on the 1991–2015 China Health and Nutrition Survey (CHNS). In total, 5,289 children were eligible for the analysis, including 3,684 rural children and 1,605 urban children. The generalized estimating equation was used to determine the influencing factors, and results were expressed as odds ratios (ORs) with 95% confidence intervals (95% CIs).

**Results:**

The results showed that rural children aged 7–12 and 13–17 years had lower odds of URTI than those aged 0–1 year, with OR value of 0.17 (95% CI, 0.11–0.27) and 0.12 (95% CI, 0.08–0.19), respectively. Compared with uneducated mothers, those with education level of primary school (OR: 0.59, 95% CI, 0.42–0.84), lower middle school (OR: 0.53, 95% CI, 0.38–0.73), and upper middle school and technical school (OR: 0.62, 95% CI, 0.40–0.95) were associated with the lower odds of URTI in rural children. Children, whose mothers were office workers, had 46% lower odds of URTI than those with farmer mothers (OR: 0.54, 95% CI, 0.34–0.84).

**Conclusions:**

This study found that mother's education level, children's age, and mother's occupation were significant influencing factors for URTI, which suggested the importance to improve mother's health-related knowledge and working conditions in Chinese rural areas.

## Introduction

Upper respiratory tract infection (URTI) refers to a viral infection that causes inflammation in the pharyngeal and nasal mucosa (1). URTI is one of the most frequent infections occurring in preschool- and school-aged children due to their immature sinus development and immature pediatric immune response (2). Each year, children experience 3–8 infections, and 52.6% of them are identified to have at least one type of URTI (3). Clinical manifestations of URTI in children include fever, runny nose, swelling of the tonsils, and a mild dry cough (1, 4). Although URTI is rarely fatal, it is a source of severe morbidities and causes some complications, thereby increasing medical costs and bringing a considerable economic burden (5, 6). Many factors make children vulnerable to URTI, many of which are preventable (7).

URTI is geographically diverse and highly associated with the living environment (8). An Ethiopian study displays that residence can affect the prevalence of URTI, and children in rural areas have a higher risk of URTI than those in urban areas (3). The study further explores influencing factors for URTI, and some factors relating to the living environment are identified, such as residence, parents' occupation, and family size (3). In China, studies have explored influencing factors for URTI in urban children (9, 10); however, factors have not been reported in rural children, who are recognized to be disadvantaged in many aspects. In Chinese rural areas, medical resources are lacking, and primary care doctors are poorly trained, which may be quite inadequate in the prevention of URTI (11). Moreover, most laborers migrate from rural to urban settings with rapid urbanization, which leads to a large number of left-behind children (12). These children have a lack of guardianship and are at risk of malnutrition, resulting in decreased immunity that may easily cause URTI (2, 12, 13). In addition, most of the guardians of rural children are farmers and have a low education level, which leads them to pay less attention to their children's health and have a lack of awareness about healthcare (14).

Considering these, we designed a cross-sectional study to explore the factors affecting URTI in rural children in China and to explore whether the factors were different from those for urban children.

## Materials and methods

### Study design and data source

This was a cross-sectional study, and the data were extracted from the China Health and Nutrition Survey (CHNS). This survey was designed to investigate at the individual, household, and community levels key public health risk factors and health outcomes, demographic, social, and economic factors (15). The survey used a multistage, random cluster process to draw a sample of over 30,000 individuals from about 7,200 households over a 7-day period in 15 provinces and municipal cities, which substantially varied in geography, economy, health indicators, and public resources (16). The survey has been approved by the Institutional Review Committees of the University of North Carolina at Chapel Hill and the National Institute for Nutrition and Health at the Chinese Center for Disease Control and Prevention.

### Study population and data collection

A total of 5,289 participants from the CHNS (1991–2015) aged <18 years and answered the question (M24B_1) “Did you have any of these symptoms during the past 4 weeks (including today)” were enrolled in this study. Demographic data [children's age, children's body mass index (BMI), children's gender, birth order, mother's age, mother's BMI, education level, and mother's occupation] and environmental data (residence, having brothers/sisters, and birth year) were collected.

Children's BMI was divided into four groups: underweight (BMI < 5%), normal weight (5% ≤ BMI ≤ 85%), overweight (85% < BMI ≤ 95%), and obesity (BMI > 95%) (17).

Mother's BMI was divided into underweight (BMI < 18.5 kg/m^2^), normal weight (18.5 kg/m^2^ ≤ BMI ≤ 24.9 kg/m^2^), overweight (25 kg/m^2 ^≤ BMI ≤ 29.9 kg/m^2^), and obesity (BMI ≥ 30 kg/m^2^) (18).

Residence was evaluated using the question “To which type of household registration do you belong, urban or rural? (1 = urban, 2 = rural).”

### URTI diagnosis

URTI was diagnosed on the basis of the questionnaire in the CHNS database. When asked if “Did you have any of these symptoms during the past 4 weeks (including today): fever, sore throat, cough,” children or their mother answered “Yes” was diagnosed with URTI.

### Statistical analysis

The classification data were described as number and percentage [*n* (%)], and difference between groups was compared using *χ*^2^ test. The missing data were processed by deletion. Factors for URTI were explored by univariate analysis, using generalized estimating equation. Multivariate analysis was used to assess and adjust the factors for URTI using generalized estimating equation. The results of the univariate and multivariate analyses were expressed as odds ratios (ORs) with 95% confidence intervals (95% CIs). Data were analyzed using R 4.1.1 (Institute for Statistics and Mathematics, Vienna, Austria). *P*-value less than 0.05 were considered as statistical significance.

## Results

### General information of rural children and urban children

A total of 5,289 children were included in this study. Of these, there were 3,684 rural children and 1,605 urban children. For children's mothers, 21% of them (*n* = 1,090) obtained education level of primary school, and 19.2% of them (*n* = 1,014) were office workers. Significant difference was found in children's age, children's BMI, children's gender, birth order, mother's age, education level, mother's occupation, having brothers/sisters, and URTI between rural children group and urban children group (Table 1).

**Table 1:** General information of rural and urban children [*n* (%)].

Variables	Total (*n* = 5289)	Rural (*n* = 3684)	Urban (*n* = 1605)	Statistics	*P*
Children's age, years				*χ*^2^ = 19.231	<0.001
0–1	357 (6.7)	246 (6.7)	111 (6.9)		
2–6	1,489 (28.2)	1,077 (29.2)	412 (25.7)		
7–12	2,075 (39.2)	1,469 (39.9)	606 (37.8)		
13–18	1,368 (25.9)	892 (24.2)	476 (29.7)		
Children's BMI, kg/m^2^				*χ*^2^ = 24.449	<0.001
Underweight	429 (8.1)	318 (8.6)	111 (6.9)		
Normal weight	3,223 (60.9)	2,300 (62.4)	923 (57.5)		
Overweight	410 (7.8)	266 (7.2)	144 (9.0)		
Obesity	1,227 (23.2)	800 (21.7)	427 (26.6)		
Children's gender				*χ*^2^ = 22.987	<0.001
Boy	2,922 (55)	2,115 (57)	807 (50)		
Girl	2,367 (45)	1,569 (43)	798 (50)		
Birth order				*χ*^2^ = 245.698	<0.001
1	2,582 (49)	1,539 (42)	1,043 (65)		
2	1,330 (25)	1,028 (28)	302 (19)		
≥3	1,377 (26)	1,117 (30)	260 (16)		
Mother's age, years				*χ*^2^ = 8.101	0.004
<35	2,625 (50)	1,876 (51)	749 (47)		
≥35	2,664 (50)	1,808 (49)	856 (53)		
Mother's BMI, kg/m^2^				*χ*^2^ = 4.002	0.261
Underweight	501 (9.5)	335 (9.1)	166 (10.3)		
Normal weight	3,698 (69.9)	2,576 (69.9)	1,122 (69.9)		
Overweight	903 (17.1)	647 (17.6)	256 (16.0)		
Obesity	187 (3.5)	126 (3.4)	61 (3.8)		
Education level				*χ*^2^ = 513.875	<0.001
Uneducated	538 (10)	423 (11.5)	115 (7.2)		
Primary school	1,090 (21)	885 (24.0)	205 (12.8)		
Lower middle school	2,122 (40)	1,618 (43.9)	504 (31.4)		
Upper middle school and technical school	945 (18)	543 (14.7)	402 (25.0)		
University or higher	594 (11)	215 (5.8)	379 (23.6)		
Mother's occupation				*χ*^2^ = 456.778	<0.001
Farmer	2,176 (41.1)	1,852 (50.3)	324 (20.2)		
Service worker	1,021 (19.3)	676 (18.3)	345 (21.5)		
Office worker	1,014 (19.2)	456 (12.4)	558 (34.8)		
Manufactory worker	804 (15.2)	534 (14.5)	270 (16.8)		
Others	274 (5.2)	166 (4.5)	108 (6.7)		
Having brothers/sisters				*χ*^2^ = 314.064	<0.001
No	1,320 (25)	663 (18)	657 (41)		
Yes	3,969 (75)	3,021 (82)	948 (59)		
Birth year				*χ*^2^ = 0.002	0.969
Before 2000	2,934 (55)	2,043 (55)	891 (56)		
After 2000	2,355 (45)	1,641 (45)	714 (44)		
URTI				*χ*^2^ = 39.312	<0.001
No	4,365 (83)	3,120 (85)	1,245 (78)		
Yes	924 (17)	564 (15)	360 (22)		

BMI, body mass index; URTI, upper respiratory tract infection.

### Influencing factors for URTI in rural children

Table 2 shows that children's age (7–12 and 13–17 years), mother's education level (primary school, lower middle school, upper middle school, and technical school), mother's occupation (office worker), and birth year were the significant factors for URTI in the multivariate analysis. Compared with children aged 0–1 year, children aged 7–12 and 13–17 years were associated with 83% and 88% decreased odds of URTI, respectively (OR: 0.17, 95% CI, 0.11–0.27; OR: 0.12, 95% CI, 0.08–0.19). Children with mothers having education level of primary school (OR: 0.59, 95% CI, 0.42–0.84), lower middle school (OR: 0.53, 95% CI, 0.38–0.73), and upper middle school and technical school (OR: 0.62, 95% CI, 0.40–0.95) had 41%, 47%, and 38% lower odds of URTI than those with uneducated mothers. Children whose mothers were office workers had 46% lower odds of URTI than whose mothers were farmers (OR: 0.54, 95% CI, 0.34–0.84). Children born after 2000 had 79% lower odds of URTI compared with children born before 2000 (OR: 0.21, 95% CI, 0.16–0.27).

**Table 2:** Exploring influence factors for URTI in rural children.

Variables	Univariate		Multivariate	
	OR (95% CI)	*P*	OR (95% CI)	*P*
Children's age, years				
2–6 (vs. 0–1)	0.85 (0.63–1.17)	0.322	0.75 (0.52–1.07)	0.108
7–12 (vs. 0–1)	0.41 (0.29–0.57)	<0.001	0.17 (0.11–0.27)	<0.001
13–17 (vs. 0–1)	0.37 (0.26–0.54)	<0.001	0.12 (0.08–0.19)	<0.001
Children's BMI, kg/m^2^				
Underweight (vs. normal weight)	0.94 (0.67–1.32)	0.728	1.08 (0.75–1.56)	0.675
Overweight (vs. normal weight)	1.11 (0.78–1.57)	0.562	1.25 (0.86–1.80)	0.239
Obesity (vs. normal weight)	0.87 (0.70–1.09)	0.230	0.86 (0.66–1.13)	0.279
Children's gender				
Girl (vs. boy)	0.97 (0.82–1.15)	0.728	—	—
Birth order				
2 (vs. 1)	1.09 (0.90–1.32)	0.396	—	—
3 (vs. 1)	0.89 (0.71–1.13)	0.343	—	—
Mother’s age, years				
≥35 (vs. <35)	0.62 (0.50–0.76)	<0.001	0.86 (0.68–1.09)	0.203
Mother's BMI, kg/m^2^				
Underweight (vs. normal weight)	1.47 (0.99–2.18)	0.056	1.11 (0.79–1.57)	0.555
Overweight (vs. normal weight)	0.92 (0.64–1.32)	0.648	1.28 (0.97–1.67)	0.076
Obesity (vs. normal weight)	0.81 (0.40–1.64)	0.561	1.00 (0.55–1.80)	0.996
Education level				
Primary school (vs. uneducated)	0.59 (0.43–0.82)	0.001	0.59 (0.42–0.84)	0.003
Lower middle school (vs. uneducated)	0.44 (0.33–0.59)	<0.001	0.53 (0.38–0.73)	<0.001
Upper middle school and technical school (vs. uneducated)	0.40 (0.27–0.58)	<0.001	0.62 (0.40–0.95)	0.030
University or higher (vs. uneducated)	0.34 (0.20–0.55)	<0.001	0.82 (0.44–1.53)	0.541
Mother's occupation				
Service worker (vs. farmer)	0.59 (0.44–0.78)	<0.001	0.84 (0.61–1.16)	0.293
Office worker (vs. farmer)	0.44 (0.31–0.63)	<0.001	0.54 (0.34–0.84)	0.007
Manufactory worker (vs. farmer)	0.65 (0.48–0.89)	0.006	0.89 (0.64–1.24)	0.497
Others (vs. farmer)	0.58 (0.35–0.96)	0.033	0.71 (0.43–1.17)	0.177
Having brothers/sisters				
Yes (vs. no)	1.11 (0.86–1.42)	0.422	—	—
Birth year				
After 2000 (vs. before 2000)	0.51 (0.41–0.62)	<0.001	0.21 (0.16–0.27)	<0.001

URTI, upper respiratory tract infection; OR, odds ratio; CI, confidence interval; BMI, body mass index.

### Influencing factors for URTI in urban children

In Table 3, children's age (7–12 and 13–17 years), mother's education level (lower middle school), and birth year were found to be the influencing factors for URTI. Children aged 7–12 and 13–17 years had 74% and 84% lower odds of URTI than those aged 0–1 year, respectively (OR: 0.26, 95% CI, 0.16–0.43; OR: 0.16, 95% CI, 0.09–0.30). Compared with children with an uneducated mother, those children with a mother obtaining education level of lower middle school had 44% decreased odds of URTI (OR: 0.56, 95% CI, 0.31–1.00). As for birth year, children born after 2000 had 68% lower odds of URTI compared with those born before 2000 (OR: 0.32, 95% CI, 0.23–0.45).

**Table 3:** Exploring influence factors for URTI in urban children.

Variables	Univariate		Multivariate	
	OR (95% CI)	*P*	OR (95% CI)	*P*
Children's age, years				
2–6 (vs. 0–1)	0.93 (0.60–1.42)	0.731	0.84 (0.54–1.30)	0.429
7–12 (vs. 0–1)	0.40 (0.26–0.61)	<0.001	0.26 (0.16–0.43)	<0.001
13–17 (vs. 0–1)	0.33 (0.21–0.53)	<0.001	0.16 (0.09–0.30)	<0.001
Children's BMI, kg/m^2^				
Underweight (vs. normal weight)	1.00 (0.65–1.54)	0.987	—	—
Overweight (vs. normal weight)	1.17 (0.79–1.74)	0.436	—	—
Obesity (vs. normal weight)	0.77 (0.58–1.03)	0.075	—	—
Children's gender				
Girl (vs. boy)	1.04 (0.82–1.31)	0.766	—	—
Birth order				
2 (vs. 1)	1.09 (0.81–1.46)	0.586	—	—
3 (vs. 1)	1.08 (0.78–1.49)	0.644	—	—
Mother’s age, years				
≥35 (vs. <35)	0.49 (0.38–0.63)	<0.001	0.85 (0.61–1.19)	0.342
Mother's BMI, kg/m^2^				
Underweight (vs. normal weight)	1.16 (0.83–1.63)	0.393	1.40 (0.92–2.13)	0.120
Overweight (vs. normal weight)	1.00 (0.77–1.30)	0.990	0.98 (0.67–1.42)	0.898
Obesity (vs. normal weight)	0.60 (0.33–1.08)	0.089	0.98 (0.46–2.07)	0.957
Education level				
Primary school (vs. uneducated)	0.84 (0.47–1.48)	0.540	0.83 (0.46–1.51)	0.550
Lower middle school (vs. uneducated)	0.61 (0.35–1.04)	0.071	0.56 (0.31–1.00)	0.048
Upper middle school and technical school (vs. uneducated)	0.65 (0.38–1.12)	0.123	0.58 (0.31–1.09)	0.091
University or higher (vs. uneducated)	0.51 (0.29–0.89)	0.017	0.51 (0.25–1.02)	0.057
Mother's occupation				
Service worker (vs. farmer)	0.62 (0.42–0.93)	0.020	1.16 (0.75–1.80)	0.506
Office worker (vs. farmer)	0.69 (0.48–0.99)	0.042	1.42 (0.85–2.35)	0.178
Manufactory worker (vs. farmer)	0.89 (0.58–1.36)	0.584	1.43 (0.91–2.25)	0.125
Others (vs. farmer)	0.64 (0.36–1.14)	0.129	1.08 (0.58–1.99)	0.810
Having brothers/sisters				
Yes (vs. no)	1.02 (0.79–1.31)	0.892	—	—
Birth year				
After 2000 (vs. before 2000)	0.72 (0.56–0.92)	0.009	0.32 (0.23–0.45)	<0.001

URTI, upper respiratory tract infection; OR, odds ratio; CI, confidence interval; BMI, body mass index.

## Discussion

Evidence has shown that URTI was one of the most common diseases among children in developing countries, especially in rural areas (2, 3, 19). The main objective of this study was to investigate factors affecting URTI in Chinese children in rural areas. In this study, we included a total of 5,289 participants and found that children's age, mother's education level, and mother's occupation were significant influencing factors for URTI in rural children. Children's age and mother's education level were factors affecting URTI in urban children.

Previous studies have indicated that a mother's education level was associated with the prevalence of many diseases, including respiratory diseases (20, 21). In this study, we found that rural children whose mothers had an education level of primary school, lower middle school, upper middle school, and technical school had lower odds of URTI than children with an uneducated mother. An earlier study in rural Ethiopia has reported the same finding (22). This may be explained that having education improved mothers' basic health knowledge, which allowed them to identify the disease early and use healthcare facilities more effectively (23). Moreover, education may educate the mother's ability to communicate with health professionals, follow treatment advice, and keep their living environment clean (22). In addition, educated mothers may have more power over their children's health decisions (22). No significant difference was found in mothers who had an education level of university or higher degree. This may be explained by the fact that the sample size of this population was relatively small in this study. The findings indicated that the government should spread children’s health-related knowledge to uneducated women through mass media, community-based interventions, and lectures. Previous studies have confirmed the effective application of these methods in improving health-related knowledge (24, 25).

Regarding children's age, the odds of URTI in children aged 7–12 and 13–17 years was lower than that in children aged 0–1 year, and no significant difference was observed in children aged 2–6 years. This is quite similar to the study reported by Kansen et al. (7) The possible reason was that sinus development and immune system in children above 7 years were maturing to prevent virus infections.

Studies in developing countries have reported the association between mother's occupation and respiratory infections (21, 26). This study found that having mothers as office workers was significantly associated with the reduced odds of URTI compared with mothers as farmer among Chinese children in rural areas. There were some possible reasons for this. The working environment of office worker mothers was cleaner and more hygienic than farmer mothers, which decreased the exposure to pollutants and viruses (26). Moreover, office worker mothers had regular commutes and had more time to feed and take care of their children (26). Evidence has displayed that a mother's feeding contributed to strengthening children's immune system, which was vital to prevent infections, including URTI (27, 28).

The data show that a mother's occupation was an important factor influencing the prevalence of rural children's URTI, but it showed no statistical significance in urban children. The possible reasons were that urban children could get better care from the family and the medical security system was relatively complete in the urban areas, which decreased the influence caused by mother's occupation. In China, children's medical and health resources between urban and rural areas were unevenly distributed (29). Children's medical service institutions ranked from high level to basic level were children's specialized hospitals, pediatrics department of general hospitals, maternity and childcare institutions, and primary healthcare institutions (30). Most of the medical talents worked in specialized children's hospitals and pediatrics department of general hospitals in urban areas (30). The primary healthcare institutions in rural areas were not only short of specialized pediatricians but also lacked vocational skills training for such personnel, resulting in a lack of capacity to treat children's diseases (30). In addition, compared with rural children, urban children were cared for by more people. They were usually taken care of by grandparents and parents, and families with high incomes may employ babysitters for children's healthcare (31).

In this study, we focused on the rural children in China and first explored the factors affecting URTI in this population. Based on the findings, we recommended that the government should provide mothers in rural areas with proper education and health-related knowledge through mass media and lectures. Moreover, community healthcare groups should be set up in rural areas to promote childcare and sanitation and to provide drug supplements to help decrease the odds of URTI. In addition, the government should improve the mother's working environment and normalize working hours.

There are some limitations to this study. First, URTI is diagnosed according to the questionnaire rather than clinical examination results, which is prone to reporting bias. Second, the data were extracted from the CHNS, which may cause some potential valuable factors not recorded in the database. Third, fathers may also play a role in the URTI of their children. The data regarding fathers were missing in the included sample, so we cannot explore the impact of fathers on children's URTI. Future studies are needed to collect data on fathers to further explore the influencing factors for children's URTI in Chinese rural areas.

## Conclusion

The study found that children's age, the mother's education level, and the mother's occupation were significant influencing factors for URTI in Chinese rural children. Our findings suggested that health-related knowledge and working conditions should be improved for uneducated and farmer mothers in Chinese rural areas by mass media, community-based interventions, and lectures.

## Data Availability

Publicly available datasets were analyzed in this study. These data can be found here: CHNS repository, https://www.cpc.unc.edu/projects/china/data/datasets/index.html.
